# Let There Be Light

**Published:** 1887-06-04

**Authors:** 


### "LET THERE BE LIGHT."

"IN the beginning God created the heavens and the earth. And the earth was without form, and void. And the Spirit of God moved upon the face of the waters. And God said, 'LET THERE BE LIGHT': and there was Light." Such, according to the most venerable of all faiths and philosophies, was the beginning of all things. First darkness, chaos, confusion, unloveliness. Intelligence looks upon the desolation, and sends beams of light from its own eyes to the remotest bounds. With light begins order, followed by progression, and then by utility, economy, beauty. All human institutions have their origins more or less in the same way. Religions, empires, societies, charities great and small,—they begin, so to speak, out of sight: they are too feeble and insignificant to be noticed. They grow and expand in a confused and uncertain kind of way. There comes a time in the history of them all when light, order, and efficiency reign in the place of chaos. If to any institution such a time does not come, that institution is foredoomed to failure and extinction.

Human beings are essentially creatures of intelligence and order. The lowest savage does not live in trees, or even in caves and rocks, if he can help it: he builds for himself a planned and ordered hut. In the course of ages the descendant of the same savage erects a palace and a cathedral. It is the primal instinct of order which animates them both. To the typical man, confusion and anarchy are hateful and intolerable. They are the parents of all that is wasteful, inefficient, and unlovely. Such a man can no more resist the impulse to turn the chaos into cosmos than the budding leaves can withstand the compelling forces of the spring. God's creatures must yield to the natures He has bestowed upon them; and the strongest compulsion of the highest kind of man is towards order and perfectness.

The hospital system is long past its beginnings: the days of its darkness and infancy are over: simplicity has given place to complexity. Like a living creature of high development it has urgent and various needs: needs experienced by many similar organisations, and for the satisfying of which it must come into the arena of conflict, and obtain by its own superior fitness those things which are essential to its existence. Intelligence, strength, skill, adaptability—all are now in immediate and daily request; and if these be not forthcoming the hospital system will go the way of the vast empires of the East, or of those monsters of olden times of whom nothing but the bones remain.

The kingdom of charity is a voluntary association the subjects of which are members by their own choice. Neither subscriber nor patient, doctor nor nurse, chaplain nor lady visitor, governor nor hall-porter, is there by compulsion. Any one of them can expatriate himself at a moment's notice, and of his own free will. But though voluntary, it is in no sense a small kingdom. Not one of the Greek states in the zenith of its power was so numerous, so wealthy, or so powerful. The great Queen Elizabeth, with her greater Chancellor Burleigh, could not command the resources which the hospital system now commands.

The voluntary nature of this kingdom is one of the chief sources of its strength and of its peculiar unity. Men and women are drawn to it by certain instincts which are the common possessions of mankind. Pity, divine pity; charity, still more divine,—these are the magnets which attract men to such a common centre as the hospital; these the sacred fires which, for a time at least, burn up the dross of conventionality, political hatreds, religious animosities, social inequalities, and fuse together into one divinely-human gold all sorts and conditions of pitying and sympathising men.

This aspect of the hospital system as a centre of unity, a force of cohesion in society, a check to disintegration, is worthy of much more consideration at the hands of statesmen and reasonable patriots of all classes than it has ever received. Disintegration means strife and hatred, violence and robbery, poverty and misery, for the helpless and the weak. None but the base have anything to gain by the violent disruption of the established order: least of all may those hope to profit by such disruption who are at the opposite extremes of the social scale. The rich and the great hold their position, not by the strength of their arm or the wisdom of their brain, but by the interaction of all those forces which tend to the maintenance of social equilibrium. Any violent catastrophe, such as other countries are by no means unfamiliar with, might topple them in a moment from their unstable elevation, and bring them to the level of the general mass of mankind. They, if they are wise, will give thoughtful and constant heed to all those institutions which make for unity, peace, and brotherhood. No mightier force exists of this nature than the hospital system. The wealthy and the leisured will not only consult their better feelings, but also their very existence in their present condition, by looking well to the prosperity and efficiency of hospitals. Though many of them as individuals are beyond all praise for their steady and constant benevolence as a class, they might do very much more. If they would diminish their expenditure on vain shows, and on foolish, sometimes even wicked amusements, they would have more time, more health, and more money to spare for deeds of charity and mercy.

But neither have the toiling masses anything to gain by violent political upheavals. To them also it is of prime importance to preserve the continuity of social order. When revolution is rife trade languishes, manufactures are suspended, and labour becomes unprofitable. Then dark days dawn for the man who has no stored savings wherewith to meet the emergency. To him the orderly progress of business is not merely comfort and safety, but the very means of life. Whoever can afford to wait the advent of brighter and more peaceful days, he cannot But the workman too often gives no thought what ever to such considerations. Why should not he open his eyes and look around and ahead as well as other men? His life and the lives of wife and children are surely as dear to him as to others. He, too, should consider what strengthens ordered freedom, general prosperity, and the public weal; and should learn to foster and maintain according to his measure all those institutions which make for peace and concord. The poor man has done little hitherto for hospitals; he has not been asked to do much. The time has now arrived when the hospitals can no longer do without his aid. By combination and co-operation what might not the workmen accomplish? Where are their leaders? Why do they not lead them in the direction of building up and strengthening those institutions which are the only arks of safety the workman has when the waves of sickness and sorrow threaten to engulf him?

What may be called the carnival, the annual festival of the hospital world, is close at hand. The Hospitals Week is upon us: the Saturday and Sunday are all but here. We cannot with truth say we are proud of what has been done in previous years, either on the Saturday or the Sunday. We are not proud, but humiliated rather. The rich and the poor both give too little. Neither the rich man nor the rich woman, neither the poor man nor the poor woman sacrifices a luxury for the hospital. Of their abundance, of their superfluity, they will all spare a little, but not of their necessity. At least ten times the amount usually collected on the Saturday and Sunday might be contributed without real injury to anybody. Can the public be shamed or persuaded by any means to give the whole question a new consideration? At the present time the Queen's Jubilee is making thoughtful men consider the advantages of settled government and established institutions, and causing them to take a firmer and more intelligent grip of those things which are seen to promote the general well-being. We have no institutions of any kind which contain fewer elements of harm, and more of good, than medical charities. They are unmixed blessings to all connected with them, to those who maintain them, and to those for whom they are maintained. If any man wishes to do deeds which are purely and absolutely good—good in themselves, good in their results, good for all men and good for ever, let him according to his utmost ability come to the help of the hospitals.

### THE HOSPITALS WEEK.

